# Exploring the barriers to Pap smear test in Iranian women: a qualitative study

**DOI:** 10.1186/s12905-021-01428-9

**Published:** 2021-08-06

**Authors:** Tayebeh Marashi, Seyed Fahim Irandoost, Javad Yoosefi Lebni, Goli Soofizad

**Affiliations:** 1 School of Public Health and Safety, Shahid Beheshti University of Medical Sciences, Tehran, Iran; 2 Department of Public Health, School of Health, Urmia University of Medical Sciences, Urmia, Iran; 3 Health Promotion Research Center, Iran University of Medical Sciences, Tehran, Iran

**Keywords:** Cervical cancer, Pap smear, Barriers, Qualitative study, Iran

## Abstract

**Background:**

Cervical cancer is one of the most frequent types of cancer in females. The Pap smear is one of the most essential ways of diagnosing and screening for this malignancy, and any failure can be caused by a number of causes. The current study sought to investigate barriers to Pap smear in Iranian women.

**Method:**

This qualitative content analysis study was conducted in Iran in 2019. Data was gathered through focus groups and individual semi-structured interviews with 32 women and health professionals. The interviewees were chosen using a combination of purposive and theoretical sampling. The data was then analyzed using the content analysis approach developed by Graneheim and Lundman. Guba and Lincoln's criteria for establishing trustworthiness were explored.

**Results:**

Data analysis resulted in the identification of four primary categories, seventeen subcategories, and 186 original concepts. The main categories include weakness of health system, difficult accessibility, low health literacy, and socio-cultural factors.

**Conclusion:**

By informing women about the necessity and importance of Pap smear, providing the conditions, facilities, and equipment to facilitate the testing process, and paying more attention to cultural and social factors in cervical cancer and Pap smear planning, interventions, and policies, barriers to Pap testing can be eliminated.

## Background

Cervical cancer is the world's second-biggest cause of mortality from cancer in women [1]. The disease is predicted to affect 11.7% of women worldwide [2]. Each year, about 500,000 incidences of cervical cancer are recorded, with 274,000 deaths as a result [3–5]. However, less developed regions account for 87% (n = 230,200 cases) of cancer mortality [6, 7] with Asia accounting for 50% of cases [8]. Cervical cancer is reported to occur at a rate of 0.36 to 3.73 per 100,000 individuals in Iranian women [8].

Early marriage, having intercourse before the age of 18 years, a history of multiple marriages (having several sexual partners), a history of many pregnancies, smoking, and sexually transmitted illnesses such as the AIDS virus are all risk factors for cervical cancer [9–11]. Human papillomavirus (HPV) strains 16 and 18 are the most common risk factors for cervical cancer [12, 13]. The incidence of this cancer rises after the age of 30 and peaks between the ages of 65 and 69. The average age of a woman diagnosed with cervical cancer in Iran is 52.2 years [3].

Cervical cancer can be avoided in two ways: first, by vaccination of teenage girls aged 9 to 13 years against HPV infection, and second, by a cervical screening of women over the age of 21 [14]. A Pap smear (PS) can detect cervical cancer and should be performed every three years by sexually active women aged 21 and up [15–17]. Cervical cancer often grows slowly and over time, but the consequent death rate will be lowered only if the illness is detected early and treated effectively [18]. Given an increase in the usage of screening programs, the incidence of cervical cancer has decreased by more than half in the previous three decades [17]. Cervical cancer screening coverage in underdeveloped countries is on average 19%, which is lower than in developed nations (63%) [19, 20]. In Iran, more than half of women have not taken the PS exam, with the proportion being substantially higher in impoverished and marginalized areas [21, 22].

Despite the fact that the PS test is a simple, inexpensive, and relatively reliable method of diagnosing cervical cancer, most women do not have it performed despite knowing how important it is [23], which could be due to a lack of awareness of the need for testing, fear of contracting cervical cancer, fear of painful tests, lack of access to health centers, and embarrassment [21, 24, 25]. According to Campbell et al. (2012), socioeconomic status, amount of education, ethnicity, and membership in a minority group all have an impact on PS testing [26]. Culture also influences the performance of health-protective behaviors (HPBs) [27, 28]. Chang et al. (2013), for example, revealed that a high incidence of cervical cancer in Chinese women was attributable to not undergoing PS testing because they considered it embarrassing conduct and felt it was associated with promiscuity and sexual immorality [28]. Del Carmen and Avila-Wallace (2013) also stated that because of deeply rooted cultural concerns, Latina women in North America avoided PS, resulting in an increase in cervical cancer and consequent mortality [27].

According to a review of literature, failure to do a PS is influenced by a number of factors that vary according to the social and cultural setting. In this regard, it is vital to explore these phenomena in Iranian society, which has its own set of values, norms, and conditions, and because the majority of relevant research is quantitative and experimental [29–31], qualitative research is required. As a result, the current qualitative study sought to investigate the barriers to PS test among Iranian women.

## Methods

### Design

This is a traditional qualitative content analysis research. The qualitative technique focuses on comprehending the complexities and subtleties of the phenomenon under investigation, and the researcher assures sustained participation [32, 33]. In conventional content analysis, categories are extracted directly from textual data and the researcher will gain a deeper understanding of a phenomenon [34, 35].

### Setting and participants

In 2019, the study population included women, health staff, obstetricians, and gynecologists, and the research site was Oshnaviyeh County in northwestern Iran. Data were gathered through semi-structured interviews and focus groups with 32 women who met the following inclusion criteria: marital status, willingness to engage in research, capacity to convey experiences and inner sentiments, and knowledge of the topic matter. Health professionals such as physicians, obstetricians, and health center workers were also sampled and questioned. The interviews took place at the participants' homes, the researcher's home, offices, and health clinics.

### Data collection

After receiving the code of ethics (IR.SBMU.RETECH.REC.1398.281) from Iran's Shahid Beheshti University of Medical Sciences, data were collected by the corresponding author, who is a PhD student in health education and is familiar with the principles of qualitative research, as well as a native resident of the study area, using a guide questionnaire completed in the participant's native language. The respondents were chosen using purposive sampling, and theoretical sampling was utilized to calculate the number of participants, the location of the needed data, and the study path. This sort of sampling, with the greatest diversity, provides for a more in-depth investigation and analysis of the nature and numerous features of the phenomena under consideration. In this regard, attempts were undertaken to create the greatest possible diversity in data. Women with various economic, social, and demographic features were chosen as samples (such as age, level of education, job, economic status, etc.).

Prior to the interviews, all of the authors prepared the general interview questions in two face-to-face meetings and one online session, as well as a question guide (Table 1). It should be emphasized, however, that the order of the interview questions was varied for each participant, and extra exploratory questions were asked in proportion to the responses supplied. In terms of group talks, the researcher met with participants in person two days before the aforesaid conversations. They were also informed of the discussion's goals and topic. The group conversation began with greetings. The researcher then introduced himself/herself and his/her research helper. The researcher then offered a quick overview of the topic of discussion as well as the study objectives, general regulations, and the session. The researcher also acquired verbal authorization from the participants to record the session material, and they were told that the session duration was limitless and would continue as long as the participants were agreeable and the conversations were in accord with the researcher's objectives. The participants were then invited to identify themselves to attempt to develop an intimate contact and break the ice. The researcher next asked questions concerning hurdles to a Pap smear, which extended the group conversation. The researcher began by asking both particular and generic inquiries. At the end of the conversation, the last question was posed to elicit open and candid responses, such as, "Do you think there is anything else connected to the issue that needs to be covered in this session?" The researcher also offered a summary of the key topics raised throughout the group discussion to ensure the accuracy of the material and to allow participants to voice any other silent ideas. The participants choose the time and location of the interviews. All interviews were taped with the participants' prior permission. In addition, none of the interviewees backed out.

**Table 1:** Checklist for questions of the interview guide

No	Questions
1	What are your thoughts on the PS test and its significance?
2	What do you know about the PS test?
3	Why do you believe women do not take this test?
4	How much pressure does society, family, and relatives put on women to take this test?
5	Has the healthcare system made it simple for women to take this test?

To adhere to ethical issues, participants were informed of the study's aims and their voluntary involvement in the research at the start of each interview. Throughout the study, individuals were guaranteed the confidentiality and anonymity of their information and informed written agreement was collected from all participants. After receiving their consent, interviews were done with each participant separately and recorded on tape.

The theoretical saturation of a category is the criterion for determining when to cease sampling the various groups relevant to that category. In qualitative research, theoretical saturation occurs when the continuation of interviews no longer aids in the generation of new data and all codes are repeated. As a result, the researcher chooses to halt the interview [36]. As a result, 32 participants were interviewed: 17 individuals and three focus groups (5 people per group). Each interview and focus group lasted around 45 and 90 min, respectively.

### Data analysis

The second and third authors of the study completed the data analysis utilizing Graneheim's and Lundman's methods [37–40] and MAXQDA2018. The initial stage was for the researcher to type the interview text in the Microsoft Word 2017 program immediately following the initial interview and on the same day, with the assistance of other research colleagues. The interview material was carefully reviewed three times by the researchers in the second phase to gain a broad feeling of their writing. In the third stage, all of the interview materials were carefully and meticulously examined word by line to extract the initial codes. In the fourth stage, the researchers classified codes that had comparable meanings and concepts into one group and judged their importance. The codes and categories were placed in the main categories in the fifth phase, which were more broad and abstract in terms of ideas. Finally, the complete data analysis process was shared, and all of the article writers' perspectives were used in a joint session.

### Trustworthiness

Lincoln and Guba's trustworthiness criteria were fulfilled in order to assess the quality of the outcomes [41–44]. Given that the researchers were native residents of the region under investigation, as well as a female researcher and interviewer, the researchers were able to acquire the participants' trust and confidence. Continuous observation and note-taking were also investigated (credibility). During the research, a qualitative research specialist observed data collection and processing, and the results were made accessible to many participants to confirm whether the results accurately reflected their experiences, and inner sentiments, and the results were approved by them (dependability). Throughout the study, the researchers attempted to prevent personal biases by recording all perspectives and views and avoiding interfering with the data gathering and analysis process as much as possible (confirmability). Individuals who did not engage in the study were given a description of the extracted categories, and they agreed on the categories. Individuals with various socioeconomic and educational levels, marital status, and age status were also chosen (transferability).

## Results

The present study was conducted on 32 women and health centers personnel and gynecologists. The mean age of the respondents was 38.75 years and the age of marriage was 5–10 years in 40.7% of cases (Table 2).

**Table 2:** Demographic characteristics of participants

Characteristic	Groups	Frequency (%)	Mean ± SD
Age	–	32 (100)	38.75 ± 11.55
Years of marriage			
	< 5	7 (21.9)	3 ± 0.81
	5–10	13 (40.7)	7.53 ± 1.71
	> 10	12 (37.4)	17.25 ± 6
Number of children			
	No children	5 (15.7)	
	Less than 3 children	21 (65.6)	1.57 ± 0.50
	3 children and more	6 (18.7)	3.33 ± 0.51
Education			
	Illiterate	5 (15.7)	
	Under Diploma	7 (21.8)	
	Diploma	4 (12.5)	
	University education	16 (50)	
Status of employment			
	Housewife	15 (47)	
	Employed (non-health sector)	9 (28)	
	Health sector employee	8 (25)	
History of Pap smear testing			
	Yes	10 (31.25)	
	No	22 (68.75)	

Data analysis led to emergence of 4 main categories and 17 sub-categories (Table 3), each of which is investigated separately below.

**Table 3:** Categories and sub-categories of Barriers to Pap smear Test in Iranian Women

Categories	Sub- Categories
**Weakness of the health system**	
	Weakness in hardware capabilities
	Poor trust in the health system
	Training weakness of the health system
	Improper behavior of health personnel
**Difficult accessibility**	
	Weakness in hardware capabilities
	Poor trust in the health system
	Training weakness of the health system
**Low health literacy**	
	Incomplete awareness and understanding
	Misconceptions
	Believing in immunity to diseases
	Avoidance of clinical medicine
	Fear of being diagnosed with cervical cancer
**Socio-cultural factors**	
	Women's financial dependence on men
	Lack of support from men and family
	Marginalization of women's health
	Fatalism
	Embarrassment

### Weakness of the health system

The health system is comprised of different sectors that seek to serve the people and reduce the negative consequences of diseases. The components of this system can acts as a facilitator or barrier to a behavior. Based on the analysis of the findings of the interviews, some characteristics of the health system act as barriers to PS testing.

### Weakness in hardware capabilities

The first sub-category refers to weaknesses in structural equipment and medical and health facilities. Lack of hospital and medical facilities, being far away from health centers, poor access of rural centers to PS testing services, shortage of gynecologists, low quality of existing health centers and referral of health center samples to other centers for examination and experiments were among the barriers to doing or repeating the test.

Our city does not have appropriate facilities and I always prefer to go to the surrounding cities for examination and testing. There is only one laboratory in our city that is not very valid and physicians working in the surrounding cities do not accept its results at all (Participant No. 5).

If I have the budget, I will bring PS mobile facilities to the villages; some villages have even commuting problems, if there are mobile facilities, obstetricians, and equipment, the women will perform PS. (Participant No. 16).

There is also no lab in the health center complex and it is farther away, which means that the sample should be taken either by taxi or their car to the laboratory on time. Sometimes it is necessary to take the sample again. All of these cause problems for women (Participant No. 7).

### Poor trust in the health system

The weakness of the facilities and expert forces, along with the challenges and difficult conditions experienced by women, have led to a significant reduction in people's trust in the health system and its health services, which itself leads people to disbelieve the results of experiments, to consider health care professionals to be incompetent, and to ignore their diagnoses and recommendations. These women even refuse to attend training held in classes health center. Overall, people regard the healthcare system as an inefficient and unreliable system, and on the contrary, refer to larger cities with better healthcare facilities.

I don't trust the doctor and the laboratories here, and I prefer to go to a place with better facilities (Participant No. 4).

People don't really trust the doctors here. I take my kids to the surrounding cities. There is no good specialist in our city, and I can't believe the hospital and its diagnosis (Participant No. 3).

I don't really believe in the advice and diagnosis of obstetricians, and that's why I don't like to refer to them. I've even seen doctors who misdiagnosed patients, and people are consequently scared of PS test (Participant No. 21).

### Training weakness of the health system

Awareness-raising and training by the health system in various fields, especially PS testing, face major challenges. One of the missions of the healthcare centers is to provide information, but the participants’ responses show that these centers are not doing their mission well. Poor performance of the health system regarding education, performing and repeating PS tests, poor information about free test plans, low quality of health center training classes, lack of training in premarital counseling, lack of peer education and local advertising and training, and the lack of medical and educational programs in the native language (Kurdish) are among weaknesses in the health system in the field of PS testing education.

"The health center should inform us about PS test. Poor information, (training and information), is the main problem with the city's health network. It should inform women about time and place of the test and time of repeating it, but it is done very less frequently." ... however, it is better if part of this education and notification is provided in Kurdish, because some women do not understand Persian well (Participant No. 12).

There are 17 co-workers in one school. If there is effective notification on the PS test in the school, a number of people will certainly refer for it, and they can train a few colleagues and encourage other colleagues to perform PS (Participant No. 14).

### Improper behavior of health personnel

People are in touch with health personnel and doctors. Personnel’s behaviors and interaction is a key determinant of women's subsequent referrals and their willingness to perform PS. The participating women noted that bad temper, harsh reactions, and inappropriate behavior of obstetricians and gynecologists prevented them from subsequent follow-ups and referrals in some cases. This situation is exacerbated especially in the case of free screening plans when people do not actually undertake no cost. In contrast, good behavior, compassionate advice, and professional relationship with women on the part of personnel and physicians encourage and motivate them for periodic examinations and regular visits.

The behavior and treatment of obstetricians and gynecologists is also very important during examination time. The more good-tempered they are, the easier it is for them to be comfortable with them (Participant No. 2).

Some obstetricians treat us very badly, they don't greet with us and don't answer every question we ask. You get discouraged and don't refer to them anymore (Participant No. 11).

### Difficult accessibility

The second major category refers to conditions for accessing Pap smear. Difficult access and the associated challenges are a major barrier to PS testing.

### High cost of Pap smear

The most important shortcoming of pap-smear policies in Iran is expensive or at least not completely free tests, which reduces people’s willingness, but they will be encouraged to perform the test if it is free of charge. Free PS screening plans are temporary. Another issue is the lack of full insurance coverage and low frequency of testing in government hospitals and health centers. In many cases, insurance does not repay the costs of testing to the insured or pay a small amount. At the same time, many government centers do not perform the test, and some women have to go to private centers and laboratories, which require high costs.

People only pay for the lab, which is little, but if it's completely free and they don't pay for the lab, people will be much more encouraged do perform the test (Participant No. 6).

Some people don't visit due to the cost and say, “the cost of testing is too much or the insurance doesn't pay off" (Participant No. 1).

### Exhausting process of Pap smear testing

Women and families are discouraged to perform PS since it is difficult, time-consuming, and exhausting. Health and laboratory centers are usually crowded, and long queues increase people's waiting time and make them confused. These conditions cause people to go to neighboring cities and endure the hardships of commuting.

When we went for PS, which was a health center plan, it was so crowded that we got tired; there were 100 people were in line (Participant No. 1).

There's an obstetrician here, and we used to have to go to the surrounding cities, but now, although we have an obstetrician, we have to wait a long time. We have to go there only when we are free, because there is only one obstetrician (Participant No. 10).

### Challenges with access to rural areas

Another barrier to PS testing for rural women is far distance from health centers and difficult commutes. The structure of the villages in the study area, which requires hard work in agriculture and animal husbandry sectors, along with relative deprivation as compared to urban areas and the far distance from urban facilities, employment in agriculture and at the same time housekeeping and childcare, deprive women of performing the test. Also, the remoteness and difficult commutes, especially during the colder months of the year, when rural women have no heavy responsibilities, is again a barrier to screening.

Rural women always refer less frequently, and one of the reasons is that it is difficult for them to come. They don’t have the time to come to the city during the agriculture season, and it is also difficult to travel during the winter. Urban women visit more frequently (Participant No. 18).

### Low health literacy

The third main category identified from the analysis of interviews is low health literacy. Although health literacy has various components and features, the subcategories of low health literacy discussed in this section are signs of this literacy that cause women to not perform PS.

### Incomplete awareness and understanding

Knowledge and awareness are key to performing PS. A part of women were unaware of PS, its functions and performance, and positive outcomes. Lack of familiarity with the risks and symptoms of cervical cancer, unawareness of the right time and age to start PS, and useful times to repeat the test are other aspects of lack of knowledge and understanding about PS and related diseases that make women not to believe in screening and do not visit the physician before the symptoms appear.

Women have little knowledge of PS, and even most of them don't know it at all. When they refer and are asked about the disease symptoms, they know almost nothing and don't know when to refer or what to do (Participant No. 20).

I think women have no information about it. I didn't know anything about PS until I went to the doctor, and what symptoms women should pay attention to and ... (Participant No. 5).

### Misconceptions

Many women have misconceptions about disease and health, and as well as incorrect interpretation of PS, its procedure, and outcomes. Women think that cervical cancer is a hereditary disease, that cervical cancer is linked to promiscuity, and that women in the study area do not need to perform PS because of their chastity, and that they must perform the test as they get older. PS is necessary for women who have an abnormal delivery (cesarean section). The uterus of women with a normal delivery is safe and does not require screening, and PS is associated with pain and bleeding.

I had vaginal deliveries in all three times. I didn't have any problems during those years and that's why I didn't refer. I don't think PS is needed for anyone who has a normal delivery (Participant No. 7).

I'm afraid of PS. They say sampling will cause pain and cervical ulcers, and they'll remove a piece of cervical tissue for sampling. These make me not refer for the test (Participant No. 29).

PS screening is not necessary for women in our area, because our women are chaste (Participant No. 17).

### Believing in immunity to diseases

Some women believe that others suffer from the disease and disease occurs for other people and not them. This belief prevents them from visiting a doctor and not feeling the need for PS. This unrealistic optimism and belief in immunity to the disease makes women less sensitive to the disease, even when they see its unusual symptoms.

Instead of referring for the required tests, many women sometimes say, “they are not prone to the disease. They kind of say they are not prone to the disease and only others are prone to the disease" (Participant No. 16).

"I was not sensitive to disease and the test, and I thought I would not afflicted with the disease, to the extent that even when I saw things that could be a symptom of the disease, I still didn't pay attention and said,” It would be improved by itself and I didn't have to go to the doctor (Participant No. 19).

### Avoidance of clinical medicine

Instead of visiting the physician and the health system, women engage in traditional and alternative behaviors. Women practice self-medication in this regard, and they use traditional herbal medicines instead of visiting the physician. There is a kind of divergence of clinical medicine, and women do not pay much attention to the advice of physicians, relatives, and experienced people.

It's not like I don't believe in doctors and chemotherapy at all, but herbal medicine is more important to me. Herbal medicines are better (Participant No. 13).

Some women don't believe in the doctor; one of my clients said, I think every doctor is his/her own doctor and can treat him/herself!' (Participant No. 16).

### Fear of being diagnosed with cervical cancer

Fear of being diagnosed with cervical cancer is a barrier to PS. Women's fear of having a positive PS result and the consequences of being diagnosed the disease and the subsequent problems arising for women and families is a barrier to PS. This fear and phobia of being diagnosed with the disease, which is mostly due to lack of knowledge about cervical cancer and ignorance about the importance of screening and PS in secondary diagnosis and prevention, put women's physical and mental health at risk.

Many people don't perform the test due to its result. They think it's better not to know, and they have fewer troubles, are less preoccupied, and their families are less bothered. I used to think so, but not now (Participant No. 1).

I'm very scared of the doctor, the hospital, and the test. Maybe it's because my mother was hospitalized in the hospital frequently and she was very bothered until she died. I myself was very bothered during childbirth, and maybe that's why I'm so afraid of the doctor and the test, etc. lest the test result is not positive and I have a bad disease (Participant No. 3).

### Socio-cultural factors

Cultural and social issues are highly regarded by women due to their persistence, power, and influence, and any action against to accepted norms, provokes different reactions. The participating women in the present study do not have high social power and influence, and women health is not a priority due to the institutionalized patriarchy. The following subcategories are related to social and cultural issues and influence the frequency of PS.

### Women's financial dependence on men

The frequency of PS varies in working women and housewives due to financial independence and dependence. Working women are more likely to perform PS because of having higher level of outcome, and accessing richer information and scientific resources. However, housewives are less likely to perform the above test due to their lower participation in the society and, as a result, their lack of awareness and financial dependence on their spouses [45]. Structural barriers such as low household incomes are an important determinant and make men unable to provide financial support to women. If women want to perform the test, their husbands should take a day off from work on that day, especially when the person's income is not adequate, the man's desire to accompany the women decreases.

One woman said, “With the current economic situation where a person has a problem with eating, she tells herself it’s unnecessary to perform PS, and I hope nothing happens'" (Participant No. 20).

I went for PS at 5:00 a.m. to be there on time. My husband also had to close his shop for a whole day to take me there, may be took us until night. The problems and troubles are too much. By the way, I have to go to the surrounding cities to get a doctor's appointment and take the test results again, and my husband take another day off (Participant No. 6).

### Lack of support from men and family

Considering cultural issues and the relatively low level of social participation and presence of women, although PS is a women's issue, it is in fact a men-centered process; to perform the test, men’s agreement and companionship is essential, and women are not allowed to go and do it without men's permission, especially in rural areas. Many men are unaware of PS and its functions, therefore, they show little companionship and support. This lack of companionship is also seen among all family members.

It's very important for men to be aware and know about PS. Women should be accompanied during the test, and if men have more information about it, women can perform the test more easily (Participant No. 5).

Some women, even if they are aware, their families still do not care about them and do not do anything to accompany them for PS (Participant No. 6).

### Marginalization of women's health

One of the barriers to doing the test is that women’s health is not a priority. That is, attention to screening and preventive measures and periodic tests is marginalized. Considering their emotional characteristics and motherly feelings, women prefer to spend family expenses on the health of their husbands and children rather than on themselves. Women's health is marginalized due to such sacrifice, as well as family's disregard of examinations and tests related to women’s health. These issues are rooted in the culture and norms of society, and mainly linked to marginalized and weak role of women in society.

Mothers usually sacrifice themselves in the family; I neglect myself but I do not neglect my children and I spend the money for their health in many cases (Participant No. 14).

### Fatalism

Another sub-category that is rooted in social issues and religious beliefs is women's fatalism. If, on the one hand, the fear of contracting the disease causes women not to refer for screening and disease prevention, on the other hand, religious beliefs have made people and women lose their fear from contracting the disease and death in many cases. Fatalists consider themselves faithful and even welcome death.

Honestly, I know the disease from God and I believe that everything has fate and I always trust in God. If I get infected, God himself takes care of me (Participant No. 22).

### Embarrassment

Women are generally opposed to showing their private parts. They consider the PS process and its results as personal and hidden affairs, and are embarrassed to make it public. The presence of gynecologists alongside obstetricians helps to improve this process, but they also believe that private parts should not be exposed to non-mahrams. Such embarrassment is more due to women's social constraints and religious teachings rather than being an individual issue, and that women's private parts should not be seen by doctors and non-mahrams.

Honestly, I'm very shy, and embarrassment make me uncomfortable with women's examinations at all, and I don't refer until I have to do so. I'm so annoyed and embarrassed that it makes me not refer (Participant No. 2).

## Discussion

The aim of the present study was to explore barriers to performing PS among Iranian women. The results showed that four main categories of barriers to performing PS. The first barrier to doing PS was the weakness of the health system. In this regard, structural equipment and improper medical and health facilities are important findings. Ersin et al. (2013) also stated that lack of access to health centers reduced women's referrals for performing PS [21]. Shortage of health personnel and health system problems have been suggested in other studies as a barrier to PS screening [46–48]. Although there may be structural problems in any city, small towns (such as the study population) are more deprived of facilities, and the health system should pay special attention to strengthening medical facilities and equipment.

One of the interesting results related to the weakness of the health system was the poor public trust in the health system. This trust includes both facilities, doctors, and other health professionals. There is no distributive justice in healthcare, and provincial centers are in better conditions. People do not trust the facilities of smaller cities, and this highlights the issue of health justice. Special arrangements need to be made to improve this interaction and communication.

The training weakness of the health system was another barrier to PS behavior among participating women. Consistent with the results of the present study, other studies have reported the importance of recommending and educating medical and health personnel to perform PS for women [9, 49]. There is little attention to education and health in the study population, which is in line with the general policies of Iran. It is necessary to pay more attention to education and strengthening educational facilities, and to produce appropriate educational content.

Improper behavior of health personnel with clients was also another result of the present study. Andreassen et al. (2017) also found that poor communication between health providers and women, as well as discriminatory behavior of health providers reduced women's willingness to perform the test [50]. The positive attitudes of the medical staff during PS process, being cheerful, considerate, and compassionate, as well as the expression of event occurring during the above test are among the expectations of women in various studies of health professionals and referred to as a motivational factors for performing screening [51, 52]. Freijomil et al. (2019) also referred to a communication gap between women and healthcare providers as a barrier to performing PS screening by women [53]. The role of gynecologists, obstetricians, and other female staff can be a key factor in encouraging women to take the test because they are of the same gender. Also, the necessary training should be given to the health professionals and the grounds for their proper interaction with women should be provided.

The second major category included difficult accessibility to PS. The high cost of the test reduces individuals' willingness to do it. Consistent with our findings, women participating in the Bahmani et al. (2017) and Nazari (2019) studies have identified financial problems as one of the main barriers to doing the test [54, 55]. Financial constraints and economic issues related to PS screening reported in other studies have also been a barrier to performing the screening [56, 57].

Another result of the present research is that the exhausting process of PS and the challenges with access to rural areas so that people are not able to perform the test due to the crowded centers, and low accessibility, and far distance from their place of residence, especially rural residents. In a review study, Islam et al. (2017) referred to urbanization was as one of facilitators of screening [58]. Yang et al. (2019) also stated that the screening time was one of the barriers to screening among rural women in East China [45]. In another study in India, Shrivastava et al. (2013) showed that rural residence and lack of access to health centers are barriers to screening [59]. Other studies have referred to exhausting process of the test and difficult accessibility of rural areas as barriers to doing PS [60, 61].

The third major category of the present research was low health literacy. Incomplete knowledge of PS, its functions, performance, and implications were major barriers to performing the test. Numerous domestic and foreign studies have referred to low level of awareness and knowledge as one of the most important barriers to performing PS screening [49, 54, 62–66]. In the review studies of Chorley et al. (2017) and Islam et al. (2017), lack of knowledge and poor understanding of screening function and role were identified as the main barriers to screening [58, 67].

Health misconceptions were another important subcategory of the present research. Many women had misconceptions about cervical cancer and the need to perform PS. Wong et al. (2009) referred to misconceptions about cervical cancer and the painful process as a barrier to performing PS [49]. Bayrami et al. (2015) and Wong et al. (2009) referred to health misconceptions and fear of possible pain as barriers to doing PS, respectively [49, 68]. Other studies have also confirmed such beliefs as barriers to screening [9, 49, 69, 70]. One of the misconceptions, even among medical staff, was the belief in chastity or promiscuity. Chang et al. (2013) attributed the unwillingness to perform PS to the promiscuity and sexual Immorality [71]. The adoption of such a view by health personnel will affect the need to educate and encourage the covered women to perform PS.

Another finding regarding the health literacy suggests belief in unrealistic immunity and optimism among women who do not perceive themselves to be at risk for the disease. Bayrami et al. (2015) also showed that most women did not perceive themselves to be at risk for cervical cancer and therefore did not perform the test [71]. A number of studies referred to low risk perception as one of the main reasons for avoiding screening among women who have never been screened [56, 72]. This optimism should be eliminated by proper education and awareness raising.

Fear of being diagnosed with the disease prevents women from attending for a PS. The results of many studies have highlighted the fear of cancer as a deterrent to PS screening [25, 54, 66]. By informing about the benefits of Pap smears in the early detection of cervical cancer, women can be both encouraged to undertake the test and reduce their fear.

The last category refers to socio-cultural factors that were new findings in the present study and were added to previous research. It indicates that the behavior of PS test is more affected by social and cultural factors. Above all, these factors play a role in the norms, teachings, and society's cultural conditions, and social conditions are a barrier to doing pap-smear. The results showed that PS testing is a male-centered process and that women's dependence on men and lack of support from men and family is a major barrier to doing so, which is consistent with the results of other studies [56, 73, 74]. Men's awareness of cervical cancer, its causes and preventive measures is one of the facilitators of regular screening. Men's awareness of this cancer can also support and encourage women to perform screening. Therefore, just as men play a role to play in the development of this cancer, they need to have access to accurate and appropriate information, and play an active role in preventing it by accompanying women and encouraging them to perform the screening.

Results of the present research showed that health of women as center of parenting support, is not a priority for many women, which endangers their health. Disregarding women's health and health needs, as compared to other family members, was also observed in Wong et al. (2009)’s study [49].

Regarding women embarrassment as one of the results of research, religious teachings and social norms strongly prevent women from showing their private parts, even to doctors. The findings of the Chorley et al. (2017)’s study show that embarrassment is a barrier to screening among Spanish women [67]. Other studies have cited embarrassment as a barrier to doing PS [45, 49, 54, 71]. It seems that the most important thing in this regard is the presence of female doctors for examination and testing. Chorley et al. (2017) found in a study that it was important for a female doctor to perform sensitive sexual examinations, such as PS, because women felt safer and more likely to perform the screening [67]. In other words, regarding PS testing as embarrassing due to the cultural structure of the society is a barrier to doing PS. The results of many studies confirm this finding and show that many women experienced very embarrassing and worrying social and psychological aspects due to having a positive PS test result, and that people refuse to perform this test due to sexually transmitted nature of HPV virus and the subsequent stigma [56, 57, 71].

The fatalism was the last finding of the present study and women were not afraid of being at risk of the disease and subsequent death. The results of several studies have been consistent with those of the present study, and religious beliefs, such as that disease and healing are God's will, have been identified as a barrier to preventive interventions for cervical cancer [54, 69, 75]. In appreciative fatalists, the belief that cancer is a person's fate and nothing can be done to prevent it, delays and challenges the process of diagnosing and treating cervical cancer, and proper planning and interventions must be designed and implemented to correct this belief.

## Limitations

Culturally, the population study is relatively traditional and closed for women, making it difficult for women to attend public places such as parks. Therefore, it was somewhat difficult to access and interview them. Therefore, we had to conduct a number of interviews in these places with the consent of women visiting health centers and medical offices, which was not a suitable place due to being crowded and time constraints of the participants. Also, a number of interviews were conducted incompletely. The other group of interviewees, through previous acquaintances with the researcher, agreed to conduct interviews in their own homes, which led imposed pressure on participants to easily answer research questions due to the presence of other family members. On the other hand, the subject matter itself was considered by some women to be a personal matter and therefore they were not very comfortable in presenting their experiences and information. In all cases, attempts were made to provide the appropriate conditions for data collection by obtaining the consent of the participants and informing them about the possibility of withdrawing from the study at any stage of the research.

## Conclusion

The findings revealed that PS in Iranian women is influenced by a variety of variables, including weakness of health system, difficult accessibility, low health literacy and socio-cultural factors. As a result, by informing women about the necessity and importance of PS, providing the conditions, facilities, and equipment to facilitate the testing process, and paying more attention to cultural and social factors in cervical cancer and PS planning, interventions, and policies, barriers to PS testing can be eliminated.

## Data Availability

There is an ethical and legal restriction on sharing our data. According to the Autonomous Committee of Research Ethics of Shahid Beheshti University of Medical Sciences (Iran) only the researchers can have access to the data.

## Abbreviation

PS: Pap smear
